# Assessment of Blood Microcirculation Changes after COVID-19 Using Wearable Laser Doppler Flowmetry

**DOI:** 10.3390/diagnostics13050920

**Published:** 2023-03-01

**Authors:** Elena V. Zharkikh, Yulia I. Loktionova, Andrey A. Fedorovich, Alexander Y. Gorshkov, Andrey V. Dunaev

**Affiliations:** 1 Research and Development Center of Biomedical Photonics, Orel State University, Komsomolskaya 95, Orel 302026, Russia; 2 National Medical Research Center for Therapy and Preventive Medicine of the Ministry of Healthcare of the Russian Federation, Petroverigsky 10, Moscow 101990, Russia

**Keywords:** laser doppler flowmetry, wearable blood perfusion sensors, COVID-19, SARS-CoV-2, rehabilitation, blood perfusion, blood flow oscillations, wavelet analysis

## Abstract

The present work is focused on the study of changes in microcirculation parameters in patients who have undergone COVID-19 by means of wearable laser Doppler flowmetry (LDF) devices. The microcirculatory system is known to play a key role in the pathogenesis of COVID-19, and its disorders manifest themselves long after the patient has recovered. In the present work, microcirculatory changes were studied in dynamics on one patient for 10 days before his disease and 26 days after his recovery, and data from the group of patients undergoing rehabilitation after COVID-19 were compared with the data from a control group. A system consisting of several wearable laser Doppler flowmetry analysers was used for the studies. The patients were found to have reduced cutaneous perfusion and changes in the amplitude–frequency pattern of the LDF signal. The obtained data confirm that microcirculatory bed dysfunction is present in patients for a long period after the recovery from COVID-19.

## 1. Introduction

The propagation of coronavirus infection, also known as COVID-19, has caused a huge number of illnesses and deaths. To date, there have been more than 650 million confirmed cases of SARS-CoV-2 infection and more than 6 million deaths worldwide (according to the Johns Hopkins University Coronavirus Resource Center). Three years after the first reported cases of SARS-CoV-2 infection, the pandemic is still far from being over. Despite the development and widespread implementation of vaccines and containment measures, COVID-19 still has a significant impact on the lives of millions of people worldwide. Emerging evidence suggests a close link between severe clinical COVID-19 and an increased risk of its vascular complications, such as thromboembolism [1]. Approximately 40–45% of cases are asymptomatic with SARS-CoV-2, but clinical observations suggest that complications may occur even in the asymptomatic course of the disease [2].

Although COVID-19 was originally considered a respiratory disease, it has now been established that it affects multiple organs and systems, including the cardiovascular system, gastrointestinal system, brain, kidney, liver, skeletal muscle, and skin of infected patients [3,4]. Recently, there is increasing evidence of the negative impact of this disease on the microcirculatory system of the blood [5,6,7]. It is known that SARS-CoV-2 affects the microcirculatory bed, causing edema and damage to endothelial cells, affects the development of microthrombosis, and capillary blockage, and causes a variety of other negative effects [8]. The development of these disorders, in addition to the direct threat to the patient’s life and health, can also be a key factor in the development of long-term consequences of coronavirus infection, significantly reducing the quality of life of patients. Serious concerns are caused by the fact that proinflammatory status and procoagulation activity can remain in patients for a long time after the recovery [9].

Recent observations show that a fairly large proportion of patients who have recovered from a coronavirus infection subsequently suffer long-term effects of the disease [10]. These include symptoms such as weakness, breathlessness, chest, and joint pain, confusion, memory and concentration problems (so-called “brain fog”), mood changes, etc. These and other symptoms can persist for months after the disease itself and significantly reduce patients’ quality of life [11]. These disorders are referred to as “long COVID” or post-COVID syndrome. Current research is largely focused on the acute stage of SARS-CoV-2, but ongoing monitoring of the long-term effects of the disease is also necessary. In this context, the need for research into the rehabilitation of patients after coronavirus infection is clear.

There is a significant body of evidence suggesting that cardiovascular complications of coronavirus can also occur in an asymptomatic course [2], making it even more difficult to detect such complications at an early stage. This means that there will be an urgent need for both diagnostic and rehabilitative measures in the next few years for patients who have suffered from this disease. In addition, there are risks of a similar clinical outcome not only with COVID-19 but also with possible future epidemics of respiratory infections. Existing diagnostic methods routinely used in clinical practice do not allow adequate assessment of blood flow at the microcirculatory level. Currently, there is a need to develop new approaches to the diagnosis of microcirculatory disorders occurring in coronavirus infection, as well as to develop strategies for individual therapy and rehabilitation of patients after COVID-19.

Despite the widespread prevalence of the disease and the incidence of cardiovascular complications, as well as the proven extensive involvement of microvasculature in pathological processes, only very few papers have been published to date on the noninvasive assessment of blood microcirculation after COVID-19 [12,13,14].

One of the most common and applicable methods for diagnosing the state of the blood microcirculation system is laser Doppler flowmetry (LDF) [15,16]. This method is widely used in the diagnosis of complications of diabetes mellitus [17,18], rheumatic diseases [19], hypertension [20] and a number of other socially important diseases. Over the years, different modifications of the conventional laser Doppler technique had been introduced, including several attempts at developing wearable devices [21,22,23].

In the COVID-19 clinic, the main focus of research using LDF was on studying the dynamic characteristics of blood flow, including the application of functional tests. It has been shown that, during the acute phase of COVID-19, patients demonstrate a reduced vasodilatory response to local heating and reduced microvascular reactivity [24]. The correlations between microcirculatory parameters measured by LDF and laboratory test results of patients during the acute period of the disease were also analysed [25]. Another study using laser speckle contrast imaging technology demonstrated reduced vasodilation in patients with COVID-19 in response to acetylcholine and sodium nitroprusside, which persists for at least 3 months after the disease [26]. We did not find any studies in the English-language literature devoted to spectral analysis of LDF recordings in patients who underwent COVID-19. Since it is known that such analysis provides valuable diagnostic information about the state of systems regulating blood flow, including the nervous system and endothelial function, the present work aimed to fill the gaps in this area.

In this context, this work aimed to comprehensively examine the changes in blood microcirculation that occur both in the acute period of the COVID-19 disease and in the long term during rehabilitation procedures.

## 2. Materials and Methods

### 2.1. Experimental Equipment

**Figure 1:**
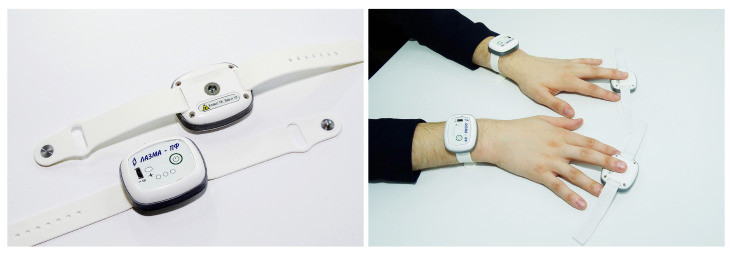
The appearance of the analysers (**left**) and the options for mounting them on the volunteer’s hands (**right**).

A distributed system consisting of 4 wireless wearable microcirculatory blood flow analysers implementing LDF method “LAZMA PF” (LAZMA Ltd, Russia; in EU/UK this device made by Aston Medical Technology Ltd., UK as “FED-1b”) was used for data recording in this study [27,28,29]. These analysers use VCSEL die chips (850 nm, 1.4 mW/3.5 mA, Philips, The Netherlands) as a single-mode radiation source. The analysers are implemented without optical fibres with direct skin irradiation from a window at the back of the instrument. This allows for avoiding fibre coupling losses as well as decreasing the movement artefacts which are common in fibre-based LDF monitors. The devices operate autonomously on internal battery power and transfer the measured signal via Bluetooth and/or Wi-Fi. The devices also have built-in motion and temperature sensors to eliminate the possible influence of motion artefacts and temperature changes on the recorded signal. When processing motion sensor data, recordings simultaneous with the subject’s movements are identified as potential sources of distortion of the LDF gram and filtered using special software. The appearance of the analysers (left) as well as the options for mounting them on the volunteer’s hands (right) are shown in Figure 1.

### 2.2. Experimental Protocol

**Figure 2:**
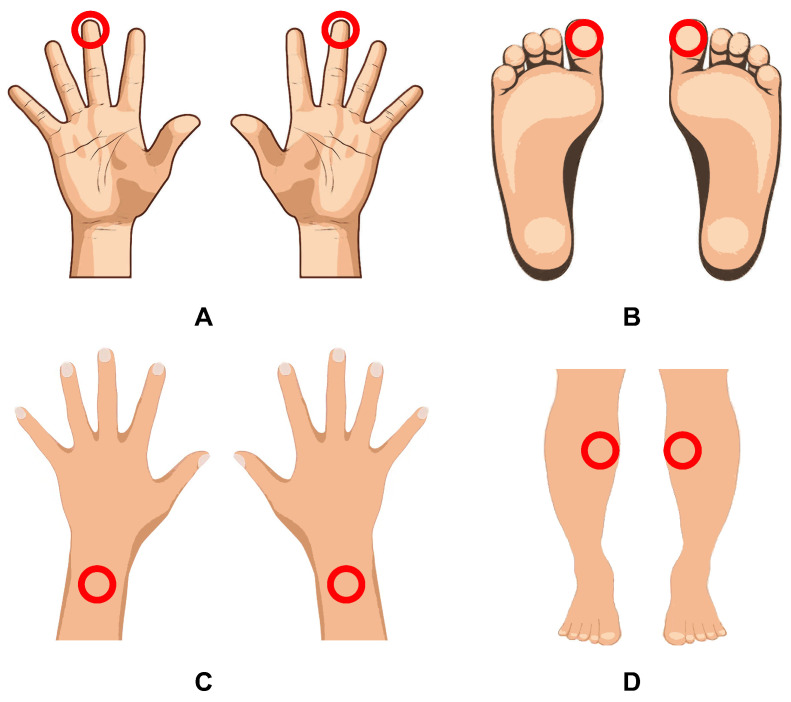
Location of the analysers on the patient’s body during the study: on fingers (**A**), toes (**B**), wrists (**C**) and shins (**D**). The device attachment positions are indicated by red areas.

The present study comprised 2 phases. The first stage involved a dynamic assessment of the processes occurring in the blood microcirculatory system during the acute period of coronavirus infection. During routine daily LDF measurements, an 18-year-old male patient was found to be accidentally infected with SARS-CoV-2 (confirmed by PCR analysis of nasopharyngeal swabs). The patient had not been vaccinated against COVID-19 prior to the study nor did he have previous experience with COVID-19. The measurements were carried out in the supine position, each lasting for 10 min. To record signals, analysers were attached to the pads of the third fingers and big toes, as well as on the dorsal surfaces of the wrists and the inner parts of the upper third of the shins. The positioning and attachment of wearable devices on the patient’s body during the study are shown in Figure 2. The measurements were taken 10 days before the onset of the disease and during 26 days after the recovery. No measurements were taken during the acute phase of the disease (7 days) because of the patient’s poor well-being. A total of more than 170 LDF signals were measured and processed over the entire study period for this patient.

**Figure 3:**
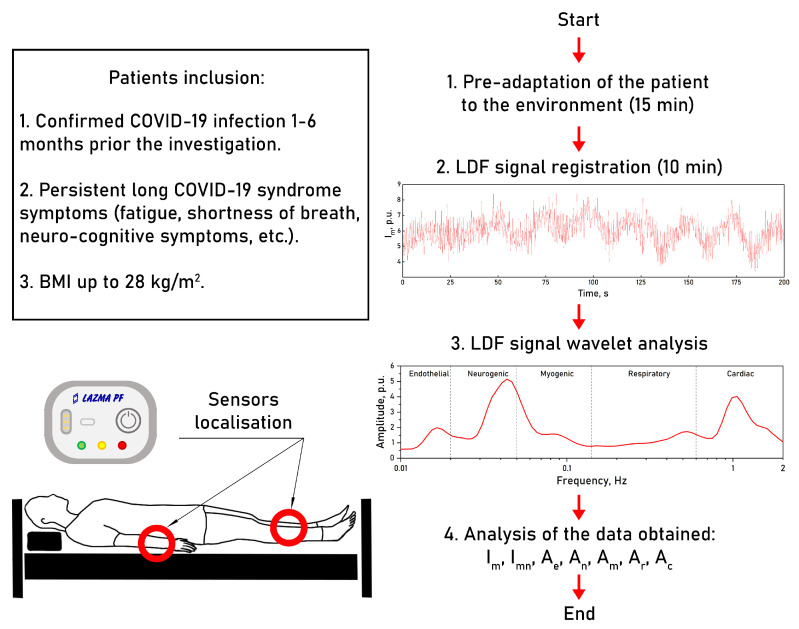
A diagram of the experimental design.

The second phase of the study involved the comparison of blood microcirculation parameters measured by LDF in a group of patients undergoing rehabilitation procedures after COVID-19 and a group of conditionally healthy volunteers with no previous history of coronavirus infection. The main group consisted of 23 subjects who had long COVID symptoms for a prolonged period of time after the recovery from an acute coronavirus infection and were undergoing rehabilitation in a private healthcare facility. Three of them had had a severe COVID-19 infection; all the other patients experienced moderate symptoms of COVID-19. Patients in the main group were measured between 1 and 6 months after the recovery. The mean age of the main group was 58±9 years. The control group included 13 conventionally healthy volunteers of a matching age who were measured in 2019 before the pandemic spread, suggesting that the volunteers in the control group had never encountered COVID-19. Volunteers with any history of cardiovascular or other serious chronic diseases affecting the circulatory system were excluded from the study. The study was conducted with the subject in the supine position in a relaxed state and consisted of a 10-min measurement of microcirculation using a wearable LDF device (“LAZMA-PF”). The analysers were attached to the dorsal surface of the forearms at a point 2 cm above the styloid process and on the inside of the upper third of the shins (see Figure 2C,D) as these points proved to be the most informative from the previous stage of the study. Figure 3 shows a diagram of the experimental design of the study.

### 2.3. Data Analysis

In the present study, the analysed parameters were the value of the index of blood microcirculation—$I_{m}$ and amplitudes of blood flow oscillations in the different frequency bands corresponding to different mechanisms of microcirculatory blood flow regulation, measured in relative perfusion units (p.u.) [30]. The endothelial ($A_{e}$) band (0.005–0.021 Hz) reflects the vascular tone regulation due to the endothelium activity, both NO-dependent and independent; the neurogenic ($A_{n}$) band (0.021–0.052 Hz) represents the influence of neural innervation on blood flow; the myogenic ($A_{m}$) band (0.052–0.145 Hz) corresponds to vascular smooth muscle activity; and respiratory ($A_{r}$) and cardiac ($A_{c}$) bands (0.145–0.6 Hz and 0.6–2 Hz, respectively) carry information about the influence of heart rate and movement of the thorax on the peripheral blood flow [31,32]. To calculate the amplitude–frequency spectra of the LDF signal, we used a mathematical apparatus of wavelet transform implemented in the software of wireless wearable analysers “LAZMA-PF”. This software performs a continuous wavelet transform using the complex-valued Morlet wavelet as the analysing wavelet.

In addition, the parameter of nutritive blood flow ($I_{mn}$), estimated by a well-known algorithm [33], was calculated. The use of this parameter makes it possible to estimate the distribution of blood flow along capillary and shunt vessels.

The statistical analysis of the data was performed in Origin Pro 2021 software. Due to the limited sample size, a non-parametric Mann–Whitney U test was used to check the statistical significance of differences. Values of p<0.05 were considered significant. The results are presented as the mean ± SD unless otherwise indicated.

## 3. Results

The first phase of the study demonstrated that COVID-19 results in changes in microcirculatory blood flow regulation mechanisms, which can be measured by assessing the spectral characteristics of the LDF signal. The results of the measurements are shown in Table 1.

**Figure 4:**
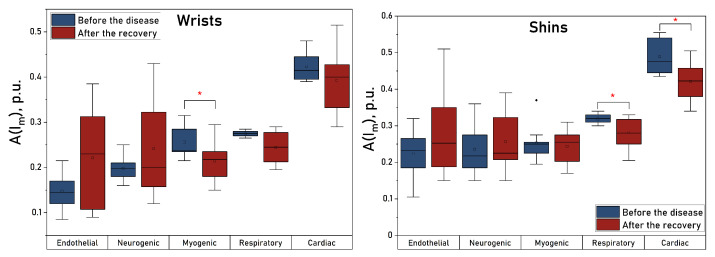
Box plots of blood flow oscillation amplitudes measured in wrists (**left panel**) and shins (**right panel**). *—The significance of the difference between the values was confirmed with p<0.05 according to the Mann–Whitney U test.

No significant changes were observed in fingers and toes in this measurement. However, there was a general trend towards a decrease in microcirculation after the disease, and also in the magnitude of the nutritive blood flow in the upper extremities. Figure 4 shows box plots of the amplitude of blood flow oscillations for the stages before and after the disease, measured in wrists and shins.

**Figure 5:**
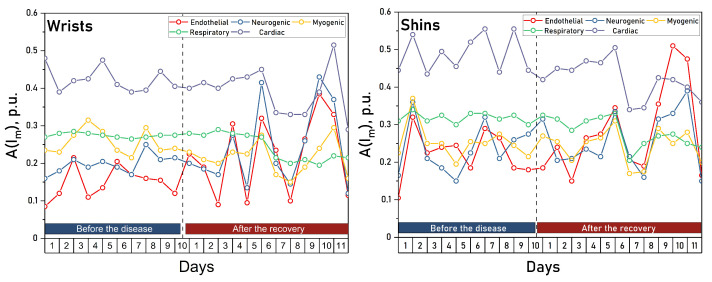
Changes in blood flow oscillations in the wrists (**left**) and shins (**right**) during the course of the disease and recovery.

A statistically significant decrease in the amplitude of myogenic oscillations was found in the arms after the disease. In the legs, a significant decrease in the amplitudes of respiratory and cardiac oscillations was observed. Similar changes can be traced in the upper extremities, but they do not reach statistically significant levels there. Figure 5 shows the dynamic changes in blood flow oscillations measured in wrists (a) and shins (b).

The figures show that COVID-19 causes high-amplitude changes in the magnitude of endothelial and neurogenic blood flow oscillations immediately after the recovery, which probably caused a high variability of these values at the “After” stage and failure to achieve a statistically significant difference in them when there is a trend for their increase after the disease. These changes are especially pronounced in the upper extremities. In the legs, there is a significant drop in the amplitude of the cardiac oscillations immediately after the disease and of the respiratory oscillations one week after the recovery, which also correlates with the results obtained in the upper extremities.

The results of the second stage of the experimental study were subsequently analysed. Table 2 presents the data obtained from the second stage of the study.

**Figure 6:**
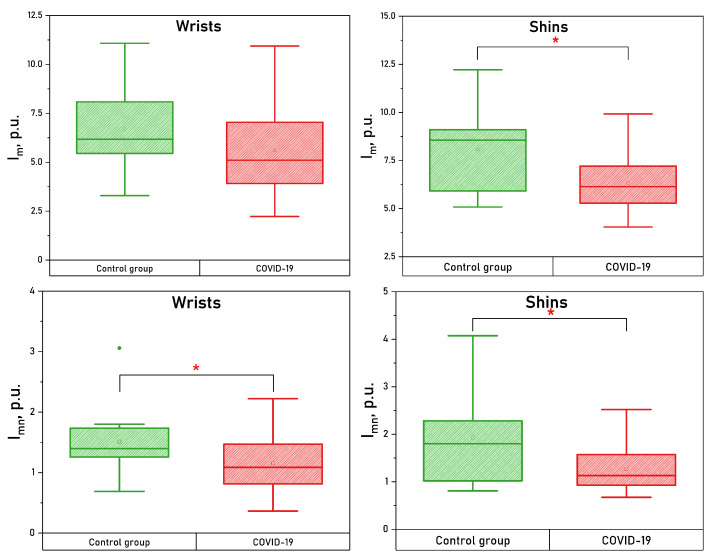
Box plots of the index of microcirculation and nutritive blood flow measured in wrists (left panel) and shins (right panel).*—The significance of the difference between the values was confirmed with p<0.05 according to the Mann–Whitney U test.

Both upper and lower extremities show significantly lower values of microcirculation and nutritive blood flow. Whisker boxes for these parameters are shown in Figure 6.

**Figure 7:**
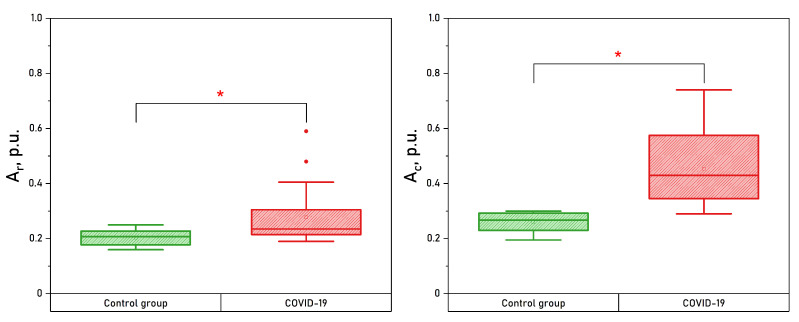
Box plots of the respiratory and cardiac oscillations measured in wrists. *—The significance of the difference between the values was confirmed with p<0.05 according to the Mann–Whitney U test.

An increase in overall oscillatory blood flow activity was also noted in both upper and lower extremities, with statistically significant differences in the neurogenic, respiratory and cardiac ranges in wrists. Whisker boxes for the respiratory and cardiac oscillations measured in wrists are shown in Figure 7.

## 4. Discussion

In the present work, we obtained experimental data, which confirm the presence of microcirculatory bed dysfunction for a long period after the recovery from COVID-19. The first part of the study, which included daily measurements of one volunteer for 10 days before his disease and almost a month after the recovery, showed that after a month the parameters did not recover to their original values.

This stage of the studies revealed a decrease in the myogenic activity of microcirculation in the upper extremities. It is worth noting that the changes in the patterns of peripheral blood flow oscillations in the post-COVID phase have not yet been studied in detail. Myogenic oscillations play an important role in the process of oxygen delivery to biological tissues [34]. A decrease in myogenic oscillations leads to an increase in the dynamic resistance of microvessels and, as a consequence, to a decrease in the nutritive blood flow. Combined with the observed decrease in neurogenic regulatory activity, this change may indicate the activation of blood flow shunt pathways. In addition, some studies show that high temperature can inhibit vasomotion [35,36], so the decrease in myogenic activity revealed in our study may be a consequence of the high body temperature of the patient during the period of the disease.

The period immediately after the recovery from COVID-19 in this study was also characterized by decreased values of respiratory and cardiac microcirculatory oscillations in both upper and lower extremities (with significant differences in legs). In this case, dynamic observations show that cardiac fluctuations are reduced immediately after the disease, and respiratory fluctuations change during the week after the recovery.

Another interesting observation of this study was the increased amplitude of endothelial oscillations in the post-COVID phase and the dynamics of these changes. Numerous studies demonstrate endothelial dysfunction as one of the main pathogenic mechanisms of COVID-19 [37,38], which can persist for more than 12 months after the recovery. Studies also show that long COVID-19 symptoms, especially nonrespiratory symptoms, are due to persistent endothelial dysfunction [39]. In our work, we observed increased amplitudes of these fluctuations both in the early stages of recovery from the disease and in the later stages (in the second phase of the study), although these differences did not reach a statistically significant level.

In a group of patients undergoing rehabilitation after COVID-19, the most interesting observation in the amplitude–frequency spectrum of the LDF signal, in our opinion, was an increase in the amplitude of neurogenic oscillations. A decrease in neurogenic tone leads to the dilation of the arterioles [40,41] and, consequently, the amplitude of cardiac oscillations significantly increases (which we can observe in our study).

The lumen size of skin arterio-venous anastomoses (AVA) is regulated exclusively by neurogenic mechanisms, so we can assume that they also expand amidst the decrease of neurogenic tone. The dilation of AVA leads to arterio-venous shunting of the blood bypassing the capillary channel, which explains the significant decrease of $I_{mn}$, a decrease of the number of functioning capillaries [13,14], reduction of perfusion ($I_{m}$) and venular overflow due to arterial blood discharge that in its turn leads to the dilation of venules [40,41] and a significant increase of the amplitude of respiratory-driven blood flow oscillations amplitude.

### Study Limitations

The present study was conducted on a small group of patients, some of whom had comorbidities, so there is no certainty that the results will be true for the broader study population. The data obtained, however, should be taken into account for the development of new diagnostic criteria in assessing the degree of microcirculatory disturbances and rehabilitation processes in recently recovered patients. There is a need for additional studies with a larger group of patients, including patients with different courses of COVID-19 (mild, moderate, and severe disease).

Despite the already three-year history of coronavirus infection and the undoubted advantages of the LDF method for diagnosing microcirculatory disorders, there are almost no studies devoted to spectral analysis of LDF signal in COVID-19 pathology. In this pilot study, we demonstrated the possibilities of laser Doppler flowmetry coupled with the wavelet analysis of the obtained signals to detect microcirculatory disorders in patients who have undergone COVID-19 that makes it a promising tool for future research and assessment of the dynamical changes in microcirculation during the recovery process.

## 5. Conclusions

The present work demonstrates the use of laser Doppler flowmetry and peripheral blood flow oscillations analysis to diagnose vascular disorders in patients who have undergone COVID-19 in their early and advanced stages of recovery. Our work demonstrated a significant increase in the amplitude of neurogenic oscillations in the upper extremities of patients undergoing COVID-19, which, as we suggest, may be a factor preceding dilation of arterioles and venules and redirection of microcirculatory blood flow from the nutritive to the shunt pathways.

The obtained data show that optical noninvasive technologies have the potential for further application, but more research is needed to fully understand the changes in the mechanisms of blood flow regulation that occur after an infection.

## Figures and Tables

**Table 1:** Results of the first part of the study.

Parameter	Wrists before	Wrists after	Shins before	Shins after	Fingers before	Fingers after	Toes before	Toes after
$I_{m}$, p.u.	4.85±0.71	4.92±0.61	8.60±1.29	8.25±1.61	21.83±1.22	21.41±1.88	19.43±3.92	17.48±2.78
$I_{mn}$, p.u.	1.99±0.44	1.78±0.33	3.10±0.78	3.14±0.78	9.56±1.64	8.70±1.69	9.15±2.99	9.71±1.78
$A_{e}$, p.u.	0.15±0.04	0.22±0.10	0.22±0.06	0.28±0.12	0.56±0.37	0.58±0.26	0.75±0.37	0.90±0.26
$A_{n}$, p.u.	0.20±0.03	0.24±0.11	0.24±0.07	0.26±0.08	0.61±0.36	0.64±0.32	0.81±0.44	1.03±0.38
$A_{m}$, p.u.	0.26±0.03	0.21±0.04 *	0.25±0.05	0.24±0.05	0.59±0.22	0.51±0.09	0.90±0.31	1.18±0.41
$A_{r}$, p.u.	0.27±0.01	0.24±0.04	0.32±0.01	0.28±0.04 *	0.45±0.03	0.42±0.03	0.44±0.08	0.43±0.05
$A_{c}$, p.u.	0.42±0.03	0.39±0.06	0.49±0.05	0.42±0.05 *	0.77±0.17	0.69±0.16	1.10±0.11	0.98±0.16

*—The significance of the difference between the values before and after the disease was confirmed with *p* < 0.05 according to the Mann–Whitney U test.

**Table 2:** Results of the second part of the study.

Parameter	Wrists CONTROL	Wrists COVID-19	Shins CONTROL	Shins COVID-19
$I_{m}$, p.u.	6.70±2.13	5.60±2.13	8.08±2.19	6.33±1.31 *
$I_{mn}$, p.u.	1.51±0.58	1.15±0.48 *	1.93±0.99	1.28±0.48 *
$A_{e}$, p.u.	0.20±0.11	0.27±0.11	0.20±0.07	0.30±0.20
$A_{n}$, p.u.	0.19±0.07	0.28±0.12 *	0.24±0.08	0.32±0.18
$A_{m}$, p.u.	0.19±0.05	0.28±0.14	0.27±0.13	0.27±0.12
$A_{r}$, p.u.	0.20±0.03	0.28±0.10 *	0.24±0.04	0.23±0.06
$A_{c}$, p.u.	0.26±0.04	0.45±0.13 *	0.39±0.06	0.54±0.31

*—The significance of the difference between the values of control group and patients was confirmed with *p* < 0.05 according to the Mann–Whitney U test.

## Data Availability

The data presented in this study are available on request from the corresponding author.
